# Detection of p73 antibodies in patients with various types of cancer: immunological characterization

**DOI:** 10.1054/bjoc.2000.1542

**Published:** 2001-01-01

**Authors:** O Tominaga, K Unsal, G Zalcman, T Soussi

**Affiliations:** Unité de génotoxicologie des tumeurs, Institut Curie, 26 rue d'Ulm, Paris, 75005; Medical Oncology department, Institut Curie

**Keywords:** p73 antibodies, p53 antibodies, tumour antigen, tumoral immune response

## Abstract

p53 antibodies have been found in the sera of patients with various types of cancer. The presence of these antibodies is generally associated with p53 accumulation in the tumour that is believed to trigger this humoral response. The recent discovery of 2 new members of the p53 family, p73 and p63, led us to study the specificity of this immune response towards the 3 proteins. Serum samples from 148 patients with various types of cancer were tested for antibodies against p73 and p63 using immunoprecipitation. 72 patients were previously shown to have p53 antibodies whereas 76 were negative. The control group consisted of 50 blood donors. p73 were detected in 22/148 (14.9%) of the cancer patients (11/72 in the group with p53-antibodies and 11/76 in the negative group). Only two sera from the control (4%) were positive. p63 antibodies were detected in only 4/148 (2.7%) of the cancer patients. Epitope mappings were performed and demonstrate that p73 antibodies are directed toward the central region of the p73 protein whereas p53 antibodies react predominantly toward the amino- and the carboxy-terminus of p53. Our results indicate that there is a specific immune response toward the p73 protein in cancer patients, a finding supported by an increasing number of publications describing p73 accumulation in tumoral cells. © 2001 Cancer Research Campaign http://www.bjcancer.com


British Journal of Cancer (2001) 84(1), 57–63
© 2001 Cancer Research Campaign
doi: 10.1054/ bjoc.2000.1542, available online at http://www.idealibrary.com on IDEAL®

http://www.bjcancer.com

### Detection of p73 antibodies in patients with various types of cancer: immunological characterization

**O Tominaga[1], K Unsal[1], G Zalcman[1,2] and T Soussi[1]**

[1]Unité de génotoxicologie des tumeurs, Institut Curie, 26 rue d'Ulm, 75005 Paris; [2] Medical Oncology department, Institut Curie

**Summary** p53 antibodies have been found in the sera of patients with various types of cancer. The presence of these antibodies is generally associated with p53 accumulation in the tumour that is believed to trigger this humoral response. The recent discovery of 2 new members of the p53 family, p73 and p63, led us to study the specificity of this immune response towards the 3 proteins. Serum samples from 148 patients with various types of cancer were tested for antibodies against p73 and p63 using immunoprecipitation. 72 patients were previously shown to have p53 antibodies whereas 76 were negative. The control group consisted of 50 blood donors. p73 were detected in 22/148 (14.9%) of the cancer patients (11/72 in the group with p53-antibodies and 11/76 in the negative group). Only two sera from the control (4%) were positive. p63 antibodies were detected in only 4/148 (2.7%) of the cancer patients. Epitope mappings were performed and demonstrate that p73 antibodies are directed toward the central region of the p73 protein whereas p53 antibodies react predominantly toward the amino- and the carboxy-terminus of p53. Our results indicate that there is a specific immune response toward the p73 protein in cancer patients, a finding supported by an increasing number of publications describing p73 accumulation in tumoral cells. © 2001 Cancer Research Campaign http://www.bjcancer.com

**Keywords:** p73 antibodies; p53 antibodies; tumour antigen; tumoral immune response

Mutations in the p53 gene are the most common genetic alterations in human cancers, including solid tumours such as colon and lung carcinomas (Soussi et al, 2000). The p53 tumour suppressor gene is located on chromosome 17p13 and encodes a nuclear 393-amino acid nuclear transcription factor which is implicated in the regulation of normal cell growth and apoptosis (Levine, 1997). Most of the known p53 gene alterations are missense mutations clustered in the evolutionarily highly conserved exons 4–8 (Soussi et al, 2000). These mutations result in a biologically inactive p53 protein that stably accumulates in the cell nucleus and can be detected by immunohistochemistry. In the absence of wild-type p53 protein, genetic aberrations are more likely to accumulate, leading to genetic instability and cell transformation.

It has been demonstrated that p53 mutations can lead to the production of p53-Ab which can be detected in the sera of patients with various types of cancers (Soussi, 2000). These antibodies recognize immunodominant epitopes localized in the amino-and, to a lesser extent, in the carboxy-terminus of human p53 (Lubin et al, 1993; Schlichtholz et al, 1994; Vennegoor et al, 1997). Antibodies specific for the central region are always poorly abundant or absent (Lubin et al, 1993). These findings are totally in accordance with the work performed in mice on the production of p53 monoclonal antibodies (mAbs). Immunization of mice with either murine, xenopus or human wild-type p53 led to the production of mAbs directed to linear epitopes localized in the amino-and carboxy-terminus of p53 (Vojtesek et al, 1992; Bartek et al, 1993; Legros et al, 1994a; Hardy-Bessard et al, 1998). MAbs specific for the central region of the protein were obtained only after a special immunization or selection procedure (Legros et al, 1994b; Vojtesek et al, 1995).

Taken together, i) the presence of immunodominant epitopes outside the hot spot region for p53 mutations, ii) the correlation between p53 accumulation (and p53 gene mutation) in tumour cells and p53-Ab responses, iii) the similarity of humoral responses in patients independent of the cancer type, and iv) the similarity of antigenic site profiles in patients and hyperimmunized animals, all suggest that p53 accumulation is the major component of the humoral response in patients with cancer. This accumulation could lead to a self-immunization process culminating in the appearance of p53-Abs. As stated above, the level of p53 proteins in a normal organism is very low, suggesting very weak (if any) tolerance to endogenous p53. Isotyping of p53-Abs has shown that they correspond mainly to IgG1 and IgG2 subclasses, while some patients exhibit a predominant IgA response (Lubin et al, 1995). Some patients also had IgM, although none had p53 IgM as the only isotype. No IgG3 or IgG4 was detected. Again, this result strengthens the hypothesis of an active humoral response to p53.

Recently, two genes referred to as p63 and p73, have been found to encode proteins that share significant amino acid identity in the transactivation domain (30%), the DNA binding domain (60%) and the oligomerization domain (37%) with p53 (Marin and Kaelin, 2000). This homology suggests that the products of this gene family may act as transcription factors but their biological functions are likely to be distinct. There is little evidence for p73 and p63 mutations in human cancer, but several consistent reports indicate that the p73 protein accumulates in the nucleus of tumour cells from different types of cancer (Ikawa et al, 1999). In our effort to study the immune response toward the p53 protein, the discovery of these new p53-related proteins raised several important questions. Do p53-Abs cross-react with p73, p63 or both? If

*Received 14 July 2000*
*Revised 18 September 2000*
*Accepted 20 September 2000*

*Correspondence to:* T Soussi so, it would be of importance to determine which of the proteins is the basis for the immune response. Furthermore, the observation that p73 is accumulated in various types of cancer suggest that it could be the target for an immune response in cancer patients.

### MATERIALS AND METHODS

### Patients

148 sera from patients with various types of cancer and 50 sera from healthy controls were tested for p63 and p73 antibodies. The distribution of cancer was as followed: 26 patients with head and neck cancer (HNC), 104 with lung cancer (LC), 17 with breast cancer (BC), one with bladder cancer. All these patients were previously tested for p53-Abs by ELISA (Lubin et al, 1995; Zalcman et al, 1998). Sera were obtained after diagnosis, but prior to any treatment. 7 ml of whole blood was centrifuged at 3000 r.p.m. for 15 min and supernatant stored at –80˚C until use.

### ELISA and immunoprecipitation procedures

All sera were previously tested for p53-Ab by the ELISA procedure (Lubin et al, 1995). p73 and p63 antibodies were screened by immunoprecipitation using $S^{35}$-labelled proteins obtained by in vitro transcription-translation using the T7 TNT-coupled reticulocyte lysate (Promega); 20 000 cpm of p53, p73 or p63 was mixed with 1 µl of serum in a final volume of 200 µl of RIPA buffer (10 mM Tris hydrochloride, pH 8.0, 150 mM NaCl, 1 mM EDTA, 1% NP40, 0.5% sodium deoxycholic acid). After 2 h of incubation on ice, 20 µl of protein A sepharose was added and the reaction was left for 20 min at 4˚C with gentle shaking. After 3 washes with 1 ml of RIPA buffer, bound proteins were eluted with Laemmli buffer and analysed by SDS-PAGE. Normal serum from a blood bank donor previously tested by ELISA and immunoblotting served as a negative control.

p53 was expressed from the construct BH11 which contains the full-length wild-type p53 cloned in Bluescribe (Stratagene) (Ory et al, 1994). p73 protein was expressed from pCMVp73 which contains the full-length wild-type p73α (Kaghad et al, 1997). p63 protein was expressed from a construct that contains only the ΔNp63γ form of p63 (Trink et al, 1998). Although it was originally named p40, we will name it p63 in order to adhere to the common nomenclature used for the p53 gene family (Levrero et al, 2000). D Caput and D Sidransky generously provided p73 and p63 constructs respectively.

### Mapping of p53, p73 and p63 epitopes

Using PCR, various regions of the cDNA encoding for p53, p63 and p70 were amplified (see delimitation of the region in Figure 1). In each case, the 5′ primer contained a T7 promoter followed by an ATG codon in frame with the coding region that was amplified. After amplification, the PCR product was directly used for in vitro transcription and translation as described above. This procedure was previously used for the epitope mapping of various p53 monoclonal antibodies (Legros et al, 1993).

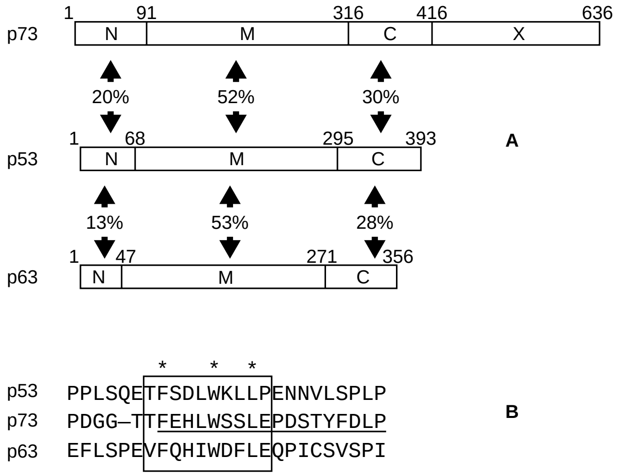

**Figure 1** (**A**) Schematic representation of the structure of p53, p73 and p63. N: amino-terminus with the transactivation domain (absent in this form of p63); M: central region with the core DNA binding domain; C: region with the oligomerization domain for the 3 proteins and the carboxy-terminus of p53 and p63: X; SAM domain which is specific for p73a. (**B**) Sequence comparison of the amino-terminus of p53, p63 and p73. This region contains the immunodominant epitopes of the p53 protein. The box delineates the mdm2 binding site of p53 with key residues indicated by an asterisk. Underlined sequence of p73 corresponds to the peptide used for immunization

### RESULTS

### p53, p73 and p63 cross reactivity

Homology between these three proteins is mainly localized to the central DNA binding domain (Figure 1A). Homology in the amino-terminus is restricted to the mdm2 binding site whereas the homology in the carboxy-terminus is localized in the oligomerization domain. We have produced several panels of mAbs toward human and xenopus p53 (Legros et al, 1994a; Hardy-Bessard et al, 1998). About 60% of these mAbs recognized an immunodominant epitope localized in the amino-terminus of the p53 that contains the binding site of p53 to mdm2 (Figure 1B). Surprisingly, none of the p53 mAbs directed toward either human or xenopus p53 cross-react with either p73 or p63 (data not shown). Furthermore, a rabbit serum raised toward the amino-terminus of p73 shows no cross-reaction toward the p53 protein (data not shown). These results suggest that the immunogenicity of these three proteins could be different.

### p73 and p63 antibodies in the sera of cancer patients

148 sera from cancer patients were tested for the presence of p73 and p63 antibodies (Table 1). 72 sera were previously demonstrated to contain p53 antibodies, whereas 76 were negative (see Materials and Methods). For the pre-screening procedure, equal amounts of labelled p73 and p63 were mixed and proceeded for immunoprecipitation with sera as described in Material and Methods. This procedure led to the detection of 22 sera reacting with p73 and 4 with p63 (Table 1). Then, each positive serum was tested separately by immunoprecipitation with p53, p73 and p63. As shown in Figure 2 and Table 1, there are clearly 4 types of sera depending on the protein recognized. Three sera were positive for the 3 proteins, 8 sera were positive for p73 and p53, 10 sera were

 © 2001 Cancer Research Campaign

**Table 1** Frequency of p73 and p63 antibodies in sera with and without p53 antibodies

| | Number of sera | p53-Abs | p73-Abs | p63-Abs[a] |
|---|---|---|---|---|
| Head and neck (HNC) | 26 | 26 | 2 | 2 |
| Lung (LC) | 28 | 28 | 6 | 1 |
| Breast (BC) | 17 | 17 | 3 | 0 |
| Bladder (UC) | 1 | 1 | 0 | 0 |
| All p53-positive[b] | 72 | 72 | 11 (15.3%) | 3 (4.2%) |
| p53 Negative (lung only)[c] | 76 | 0 | 11 (14.5%) | 1 (1.3%) |
| Healthy controls | 50 | 0 | 2 | 0 |

[a]All sera reacting with p63 also recognized p73. [b]All these sera were previously shown to have p53-Abs by ELISA. Most of them were also tested by immunoprecipitation in the present studies, confirming the presence of p53-Abs. [c]All these sera were previously shown to be devoid of p53-Abs by ELISA. Sera with p73-Abs were checked for p53-Abs by immunoprecipitation and were found to be negative.

positive for p73 and 1 serum was positive for p73 and p63. As shown in Figure 2, immunoprecipitation of p73 or p63 led to a strong signal, suggesting that the level of serum antibodies is high. Indeed, dilution experiments have shown that sera diluted to 1/50 lead to a clear positive signal (data not shown). The detection of p73-Abs in p53-negative sera suggests that this is a true immune response toward p73. This is strengthened by the observation that the frequency of p73-Abs is similar in the p53 positive and negative populations. Nevertheless, we cannot exclude that there are some antibodies that could react with the two proteins.

### Epitope mapping of p53, p73 and p63 antibodies

We have previously demonstrated that the p53 protein contains immunodominant epitopes localized in the amino-, and to a lesser extent, in the carboxy-terminus of the protein. Monoclonal or polyclonal p53-Abs are generally directed toward theses epitopes, whether from mice or humans. Using truncated protein, we have mapped the region of p73 and p63 reacting with serum antibodies. The same procedure was also applied to the p53 protein. Results are shown in Figures 3–5 and are summarized in Tables 2–4. p53-Abs reacted mainly with the N and C fragment, confirming our previous mapping experiments using either short peptides or

**Table 2** Epitope mapping of p53 antibodies in sera devoid of p73 and p63 antibodies (8 patients)

| Serum | p53 IP | p53 Epitope mapping | | | p73 IP | p63 IP |
|---|---|---|---|---|---|---|
| | | N | M | C | | |
| HNC191 | + | – | – | + | – | – |
| HNC136 | + | + | – | + | – | – |
| HNC250 | + | + | – | + | – | – |
| HNC255 | + | + | – | + | – | – |
| LC846 | + | + | – | + | – | – |
| LC991 | + | + | – | + | – | – |
| LC365 | + | – | – | + | – | – |
| LC329 | + | – | – | + | – | – |

IP: presence (+) or absence (–) of antibodies tested by immunoprecipitation. Regions N, M and C are defined in Figure 1.

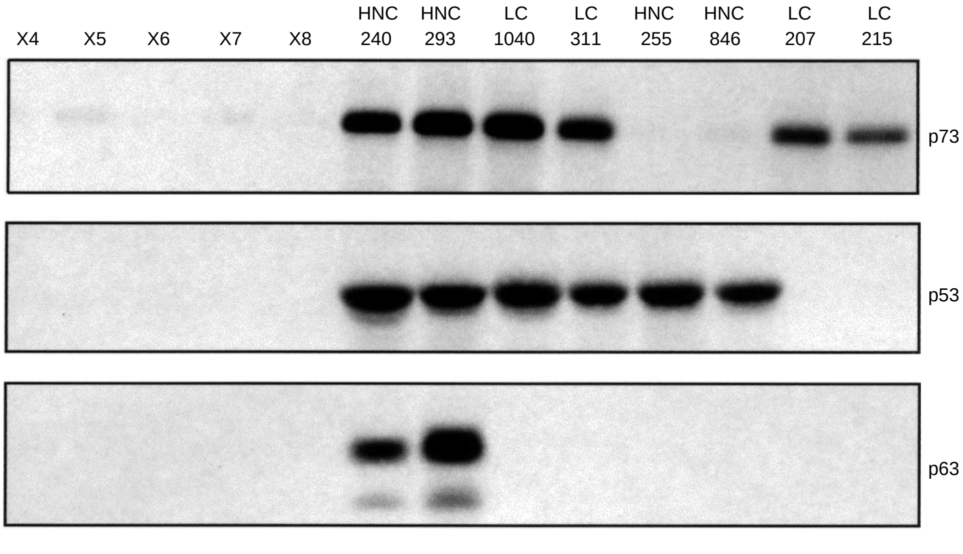

**Figure 2** Immunoprecipitation of in vitro translated p53, p63 and p73 with sera from patients and healthy donor. In vitro translated proteins were immunoprecipitated with each serum as described in Materials and Methods. X4 to X8: serum from blood donor; HNC: serum from patient with head and neck cancer; LC: sera from patients with lung cancer

*© 2001 Cancer Research Campaign*

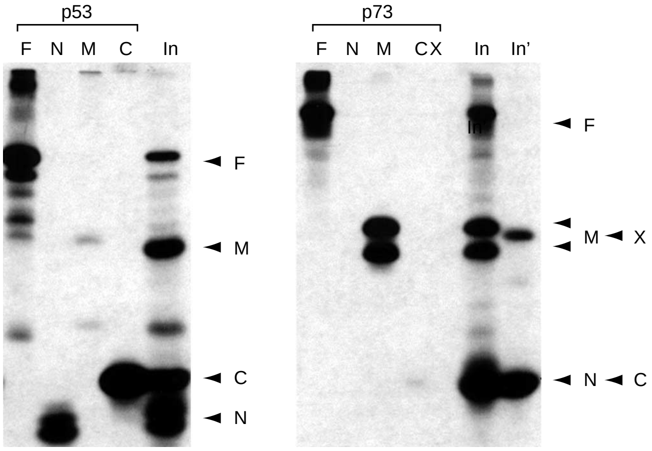

**Figure 3** Epitope mapping of serum from patient LC1040. Various regions of the p53 and the p73 protein were amplified by PCR and translated in vitro as described in Materials and Methods (see Figure 1 for the delineation of the various regions). In: Input corresponding to the various labelled proteins used for immunoprecipitation. For p73, input was loaded in two lanes (In and In′) as several products had a similar apparent molecular weight. F: full-length protein

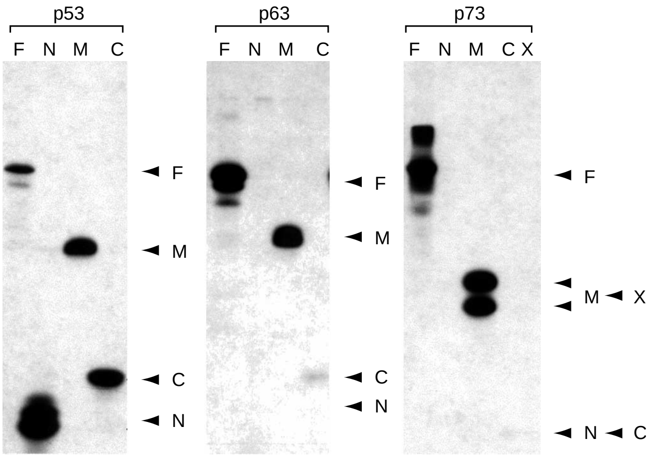

**Figure 4** Epitope mapping of serum from patient HNC293. Various regions of the p53, p63 and the p73 protein were amplified by PCR and translated in vitro as described in Materials and Methods (see Figure 1 for the delineation of the various regions). Arrowheads indicate the migration of the various proteins. F: full-length protein

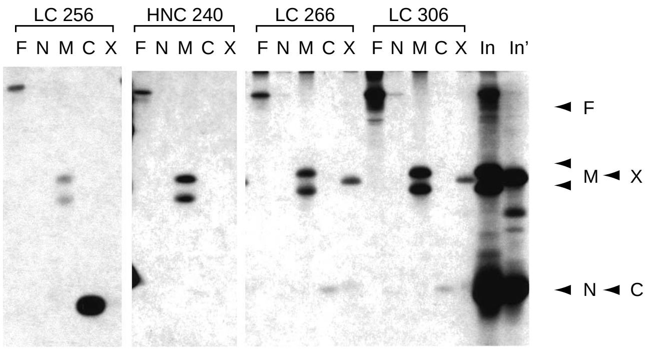

**Figure 5** Epitope mapping of 4 sera on p73 protein. Various regions of the p73 protein were amplified by PCR and translated in vitro as described in Materials and Methods (see Figure 1 for the delineation of the various regions). In: Input corresponding to the various labelled proteins used for immunoprecipitation. They were loaded in two lanes (In and In′) as several products have a similar apparent molecular weight. F: full length protein

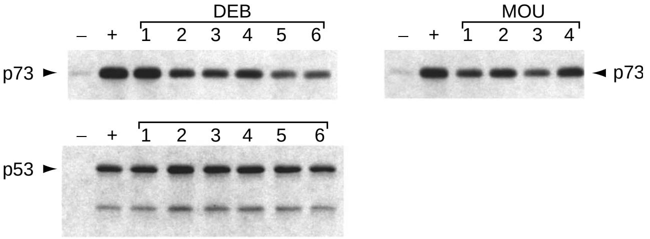

**Figure 6** Follow-up of patients DEB and MOU. Labelled p73 and p53 was immunoprecipitated as described in Materials and Methods. (–) negative control; (+) positive control. For patient DEB, lanes 1 to 6 corresponds to days 1, 26, 54, 111, 139 and 232 after diagnosis. For patient MOU, lanes 1 to 4 corresponds to days 1, 128, 288 and 383 after diagnosis. The second band below p53 corresponds to a truncated p53 produced during in vitro translation

Western blot with truncated protein (Figures 3 and 4). Some sera also contained p53-Abs that reacted with the central region of the protein (M fragment) (Figure 4 and Table 4).

Strikingly, the behaviour of p73-Abs was totally different. All sera with p73-Abs reacted with the M fragment that contains the central DNA binding region (Figures 3–5 and Tables 3 and 4). 5 sera reacted also with the C fragment that contains the

**Table 3** Epitope mapping of p73 and p63 antibodies in sera devoid of p53 antibodies (11 patients)

| Serum | p73 IP | p73 Epitope mapping | | | | p63 IP | p63 Epitope mapping | | | p53 IP |
|---|---|---|---|---|---|---|---|---|---|---|
| | | N | M | C | X | | N | M | C | |
| LC265 | + | – | + | + | – | + | – | + | – | – |
| LC207 | + | – | + | + | – | – | | | | – |
| LC215 | + | – | + | – | – | – | | | | – |
| LC220 | + | – | + | + | – | – | | | | – |
| LC247 | + | – | + | – | – | – | | | | – |
| LC256 | + | – | + | + | – | – | | | | – |
| LC266 | + | – | + | – | + | – | | | | – |
| LC281 | + | – | + | – | + | – | | | | – |
| LC286 | + | – | + | – | – | – | | | | – |
| LC306 | + | – | + | – | + | – | | | | – |
| LC330 | + | – | + | – | – | – | | | | – |

IP: presence (+) or absence (–) of antibodies tested by immunoprecipitation. Regions N, M, C and X are defined in Figure 1.

© 2001 Cancer Research Campaign

**Table 4** Epitope mapping of p73 and p63 antibodies in sera with p53 antibodies (11 patients)

| Serum | p53 IP | p53 Epitope mapping | | | p73 IP | p73 Epitope mapping | | | | p63 IP | p63 Epitope mapping | | |
|---|---|---|---|---|---|---|---|---|---|---|---|---|---|
| | | N | M | C | | N | M | C | X | | N | M | C |
| HNC240 | + | + | + | + | + | – | + | – | – | + | – | + | – |
| HNC293 | + | + | + | + | + | – | + | – | – | + | – | + | – |
| LC238 | + | + | – | + | + | – | + | – | – | + | – | + | – |
| LC311 | + | + | – | + | + | – | + | +[a] | – | – | | | |
| LC243 | + | + | + | + | + | – | + | – | – | – | | | |
| LC1040 | + | + | – | + | + | – | + | – | – | – | | | |
| LC277 | + | – | – | + | + | – | + | – | – | – | | | |
| LC437 | + | + | + | + | + | – | + | – | – | – | | | |
| BC 15 | + | – | + | + | + | – | + | – | – | – | | | |
| BC 28 | + | ND | ND | ND | + | ND | ND | ND | ND | – | | | |
| UC 338 | + | ND | ND | ND | + | ND | ND | ND | ND | – | | | |

IP: presence (+) or absence (–) of antibodies tested by immunoprecipitation. ND: Not determined (no sample remained for epitope mapping). Regions N, M, C and X are defined in Figure 1. [a] Weak positivity.

oligomerization domain and 3 with the carboxy-terminus that contains the SAM domain (Tables 3 and 4). No serum contained antibodies specific for the N fragment that contains the amino-terminus of p73. A similar result was obtained with sera containing p63-Abs, as these 4 sera interacted only with the M fragment of p63, with no antibodies toward the N and the C fragment. Sera from 4 patients (LC 238, LC 311, LC1040 and LC277), contained a high level of antibodies directed toward the M fragment of p73 that did not cross-react with the same region of p53 despite extensive homology (Figure 3 and Table 4).

### p73 antibodies and follow-up

It has previously been shown that p53-Abs can be used for monitoring the follow-up of patients during therapy (Hammel et al, 1997; Polge et al, 1998; Zalcman et al, 1998). The presence of p73-Abs was tested in a series of patients for whom serum was available during therapy (data not shown, patients diffrent from those described in Table 1). Two patients were found to have p73-Abs. One patient (DEB) had p53-Abs and the other was negative (MOU). Patient DEB had a small cell lung cancer (SCLC) and underwent a complete response to therapy (chemotherapy followed by radiotherapy) (Zalcman et al, 1998). Patient DEB was shown to have a decrease in p53-Abs during the therapy demonstrated by ELISA and which was confirmed in the present study by immunoprecipitation (Figure 6) (Zalcman et al, 1998). A similar observation was made for p73-Abs (Figure 6). Patient MOU had no p53-Abs but displayed a strong response to p73 that seemed to be constant. This patient was apparently free of cancer but displayed mycobacterium Kansasii pulmonary infection.

### DISCUSSION

p53-Abs in the sera of patients with breast cancer were first detected in 1982 (Crawford et al, 1982). Subsequent studies demonstrated that these antibodies are found in the sera of patients with different types of cancer but are absent in the normal population (Soussi, 2000). These antibodies are usually associated with the accumulation of mutant p53 protein in the tumour. Recently discovered p53 family proteins, such as p73 and p63, were shown to have significant amino acid homology and share some functions with the p53 protein. Although the eventual implication of p73 in human cancer is not fully elucidated, it is essential to evaluate the specificity of the p53 humoral response toward these two new p53 homologues.

p73-Abs were found in 14.9% of the 148 sera from cancer patient and in 4% of the 50 healthy control ($P = 0.03$, $\chi^2$ test).

The present study focused primarily on lung and head and neck patients with tumours associated with a p53 immune response. It would be of interest to test other cancer populations in order to gain a more precise evaluation of p73-Ab frequency, especially in tumours that are not associated with p53-Abs such as melanoma and brain tumours.

One of our main interests was to determine whether p53-Abs found in sera of cancer patients could cross-react with p73 or p63. The positive rate for anti-p73 antibody was similar in sera with p53-Abs and those without p53-Abs, suggesting that the p73 protein can also induce a humoral response in cancer patients.

For further characterization of these autoimmune antibodies, epitope mapping was performed using protein fragments of p53, p73 and p63. As previously shown (Lubin et al, 1993), epitopes for anti-p53 Abs are mainly located in the amino- and carboxyl-terminus of p53, whereas the majority of anti-p73 antibodies recognize the central part of the p73 protein. All p73-Abs react with the central region of p73, whereas none recognize the amino-terminus of the protein. This observation is in striking opposition with the situation observed for p53. Indeed, 4 sera (LC 238, LC 311, LC1040 and LC277), react with the central region of p73 without any cross-reaction with the central region of p53. Taken together, these results confirm the specificity of p53-Abs toward the p53 protein as none of the p73-Abs react with the immunodominant epitope of p53. Furthermore, we report for the first time that the p73 protein can elicit a specific immune response in cancer patients.

The frequency of p63 Abs is low compared to p53 or p73. They have been found only in patients with p73-Abs. Thus, it is difficult to determine whether these antibodies arise from a specific immune response toward p63 or if there is a cross-reaction of between the two proteins which share 80% homology in the central region. A recent report has described a unique syndrome in which patients had chronic ulcerative stomatosis associated with antibodies toward a 70-kDa nuclear protein (Lee et al, 1999). Cloning and sequencing the cDNA coding for this protein reveal it to be the human p63 suggesting that p63 could also be

© 2001 Cancer Research Campaign 

immunogenic, but there are no data indicating whether these antibodies also cross-react with p73 (Lee et al, 1999).

The status of p73 in human cancer is still a matter of debate. Although LOH of the p73 gene is high in several types of cancer, there is no definitive proof that the remaining p73 allele is inactivated. p73 mutations seem to be a very rare event. However, the expression of p73 has been shown to be higher in tumour tissue than in the corresponding normal tissue both at the mRNA level (Loiseau et al, 1999; Takada et al, 1999; Yokomizo et al, 1999) and at the protein level (Bjork-Eriksson et al, 1999; MacCallum and Hupp, 1999; Peters et al, 1999; Scherr, 1999; Tannapfel et al, 1999; Cai et al, 2000; Herath et al, 2000; Ng et al, 2000). The origin of such increased expression and its function in tumorigenesis is not known, but it could be involved in the triggering of this p73 immune response. Only studies combining a parallel evaluation of p73 expression and p73-Abs could elucidate this phenomenon.

Immunodominant epitopes in the p53 protein are localized predominantly in the amino-terminus of the protein. Fine mapping indicates that region 16 to 30 is highly immunogenic with 3 residues essential for antibody recognition, (residues 19, 23, 26), regardless of whether they are mAbs raised in mouse or p53-Abs from patient sera (Portefaix et al, 2000). These 3 residues have been shown to be essential for the binding of mdm2 to p53, suggesting that this region of p53 is highly accessible and localized at the surface of the protein (Kussie et al, 1996). Indeed, mAbs specific for this region compete with mdm2 for p53 binding. This region is conserved between p53 and p73, and it has been shown that mdm2 binds to p73 both in vitro and in vivo. This binding does not lead to the degradation of p73 and it is not known whether the recognition has any biological significance, or whether it occurs under physiological conditions. The observation that none of the sera with p73-Abs recognize this region despite sequence and biological homology suggests that its accessibility is different, and its biological function could be different as well.

The observation that all p73-Abs recognize the central region of the p73 protein raises several questions concerning its structural and spatial organization. The central region of the p53 protein is poorly immunogenic and only special immunization and screening procedures have permitted obtaining monoclonal antibodies directed to this domain. It has been suggested that such a hydrophobic region is very compact in the native p53 protein and buried inside the whole tetramer. One class of mutation found in human cancer changes the conformation of the protein, revealing several hidden epitopes (Legros et al, 1994b; Ory et al, 1994). Although the sequence homology between the central region of p53 and p73 is about 60%, the 2 regions behave differently. This is supported by the observation that SV40 large T antigen binds only to p53 through this central region, whereas p73 does not recognize the viral protein (Marin et al, 1998).

The present work confirms the specificity of p53-Abs found in the sera of patients with different types of cancer. The finding of specific p73-Abs unrelated to the presence of p53-Abs indicates that the status of p73 in human cancer deserves to be more carefully analysed.

### ACKNOWLEDGEMENTS

We are grateful to D Caput and D Sidransky for their gift of p73 and p63 constructs respectively. We thank Dr J. Benard and J. Bram for critical reading of the manuscript. This work was supported by grants from the Ligue Nationale contre le Cancer (Comité de Paris), Fondation de France and the Association pour la Recherche contre le Cancer (ARC). O Tominaga was a recipient of a postdoctoral fellowship from the ARC.

### REFERENCES

Bartek J, Bartkova J, Lukas J, Staskova Z, Vojtesek B and Lane DP (1993) Immunohistochemical analysis of the p53 oncoprotein on paraffin sections using a series of novel monoclonal antibodies. *J Pathol* **169**: 27–34

Bjork-Eriksson T, West CML, Cvetskovska E et al (1999) The expression of p73 is increased in lung cancer, independent of p53 gene alteration. *Brit J Cancer* **80**: 1623–1629

Cai YC, Yang G, Nie Y et al (2000) Molecular alterations of p73 in human esophageal squamous cell carcinomas: loss of heterozygosity occurs frequently; loss of imprinting and elevation of p73 expression may be related to defective p53. *Carcinogenesis* **21**: 683–689

Crawford LV, Pim DC and Bulbrook RD (1982) Detection of antibodies against the cellular protein p53 in sera from patients with breast cancer. *Int J Cancer* **30**: 403–408

Hammel P, Boissier B, Chaumette MT et al (1997) Detection and monitoring of serum p53 antibodies in patients with colorectal cancer. *Gut* **40**: 356–361

Hardy-Bessard AC, Garay E, Lacronique V et al (1998) Regulation of the specific DNA binding activity of Xenopus laevis p53: evidence for conserved regulation through the carboxy-terminus of the protein. *Oncogene* **16**: 883–890

Herath NI, Kew MC, Whitehall VL et al (2000) p73 is up-regulated in a subset of hepatocellular carcinomas. *Hepatology* **31**: 601–605

Ikawa S, Nakagawara A and Ikawa Y (1999) p53 family genes: structural comparison, expression and mutation [see comments]. *Cell Death Differ* **6**: 1154–1161

Kaghad M, Bonnet H Yang A et al (1997) Monoallelically expressed gene related to p53 at 1p36, a region frequently deleted in neuroblastoma and other human cancers. *Cell* **90**: 809–819

Kussie PH, Gorina S, Marechal V, Elenbaas B, Moreau J, Levine AJ and Pavletich NP (1996) Structure of the MDM2 oncoprotein bound to the p53 tumor suppressor transactivation domain. *Science* **274**: 948–953

Lee LA, Walsh P, Prater CA et al (1999) Characterization of an autoantigen associated with chronic ulcerative stomatitis: the CUSP autoantigen is a member of the p53 family. *J Invest Dermatol* **113**: 146–151

Legros Y, Lacabanne V, D'Agay MF, Larsen CJ, Pla M and Soussi T (1993) Production of human p53 specific monoclonal antibodies and their use in immunohistochemical studies of tumor cells. *Bull. du Cancer* **80**: 102–110

Legros Y, Lafon C and Soussi T (1994a) Linear antigenic sites defined by the B-cell response to human p53 are localized predominantly in the amino and carboxy-termini of the protein. *Oncogene* **9**: 2071–2076

Legros Y, Meyer A, Ory K and Soussi T (1994b) Mutations in p53 produce a common conformational effect that can be detected with a panel of monoclonal antibodies directed toward the central part of the p53 protein. *Oncogene* **9**: 3689–3694

Levine AJ (1997) p53, the cellular gatekeeper for growth and division. *Cell* **88**: 323–331

Levrero M, De Laurenzi V, Costanzo A, Gong J, Wang JY and Melino G (2000) The p53/p63/p73 family of transcription factors: overlapping and distinct functions. *J Cell Sci* **113**: 1661–1670

Loiseau H, Arsaut J and Demotes-Mainard J (1999) p73 gene transcripts in human brain tumors: overexpression and altered splicing in ependymomas. *Neurosci Lett* **263**: 173–176

Lubin R, Schlichtholz B, Bengoufa D et al (1993) Analysis of p53 antibodies in patients with various cancers define B-Cell epitopes of human p53 – distribution on primary structure and exposure on protein surface. *Cancer Res* **53**: 5872–5876

Lubin R, Schlichtholz B, Teillaud JL, Garay E, Bussel A, Wild C and Soussi T (1995) p53 antibodies in patients with various types of cancer: assay, identification and characterization. *Clinical Cancer Res* **1**: 1463–1469

MacCallum DE and Hupp TR (1999) Overexpression of the wild type p73 gene in breast cancer tissues and cell lines. *Cancer Res* **59**: 3257–3263

Marin MC and Kaelin WG (2000) p63 and p73: old members of a new family. *Biochim Biophys Acta* **1470**: M93–M100

Marin MC, Jost CA, Irwin MS, DeCaprio JA, Caput D and Kaelin WG, Jr (1998) Viral oncoproteins discriminate between p53 and the p53 homolog p73. *Mol Cell Biol* **18**: 6316–6324

© 2001 Cancer Research Campaign

Ng SW, Yiu GK, Liu Y, Huang LW, Palnati M, Jun SH, Berkowitz RS and Mok SC (2000) Analysis of p73 in human borderline and invasive ovarian tumor. *Oncogene* **19**: 1885–1890

Ory K, Legros Y, Auguin C and Soussi T (1994) Analysis of the most representative tumour-derived p53 mutants reveals that changes in protein conformation are not correlated with loss of transactivation or inhibition of cell proliferation. *EMBO J* **13**: 3496–3504

Peters UR, Tschan MP, Kreuzer KA et al (1999) Distinct expression patterns of the p53-homologue p73 in malignant and normal hematopoiesis assessed by a novel real-time reverse transcription-polymerase chain reaction assay and protein analysis. *Cancer Res* **59**: 4233–4236

Polge A, Bourgaux JF, Bancel E et al (1998) p53 and follow-up of colorectal adenocarcinomas. *Dig Dis Sci* **43**: 1434–1442

Portefaix JM, Thébault S, Bourgain-Guglielmetti F et al (2000) Critical residues of epitopes recognized by several anti-p53 monoclonal antibodies correspond to key residues of p53 involved in interaction with the mdm2 protein. *J Imm Methods* in press

Scherr DS (1999) Expression of p73 and Its relation to histopathology and prognosis in hepatocellular carcinoma. *J Nat Cancer Inst* **91**: 1154–1158

Schlichtholz B, Tredaniel J, Lubin R, Zalcman G, Hirsch A and Soussi T (1994) Analyses of p53 antibodies in sera of patients with lung carcinoma define immunodominant regions in the p53 protein. *Br J Cancer* **69**: 809–816

Soussi T (2000) p53 Antibodies in the sera of patients with various types of cancer: a review. *Cancer Res* **60**: 1777–1788

Soussi T, Dehouche K and Béroud C (2000) p53 Website and analysis of p53 gene mutations in human cancer: Forging a link between epidemiology and carcinogenesis. *Hum Mutat* **15**: 105–113

Takada N, Ozaki T, Ichimiya S, Todo S and Nakagawara A (1999) Elevated and biallelic expression of p73 is associated with progression of human bladder cancer. *Cancer Res* **59**: 2791–2793

Tannapfel A, Engeland K, Weinans L, Katalinic A, Hauss J, Mossner J and Wittekind C (1999) Expression of p73, a novel protein related to the p53 tumour suppressor p53, and apoptosis in cholangiocellular carcinoma of the liver. *Brit J Cancer* **80**: 1069–1074

Trink B, Okami K, Wu L, Sriuranpong V, Jen J and Sidransky D (1998) A new human p53 homologue. *Nature Med* **4**: 747–748

Vennegoor C, Nijman HW, Drijfhout JW et al (1997) Autoantibodies to p53 in ovarian cancer patients and healthy women: A comparison between whole p53 protein and 18-mer peptides for screening purposes. *Cancer Lett* **116**: 93–101

Vojtesek B, Bartek J, Midgley CA and Lane DP (1992) An immunochemical analysis of the human nuclear phosphoprotein-p53 – new monoclonal antibodies and epitope mapping using recombinant-p53. *J Immunol Methods* **151**: 237–244

Vojtesek B, Dolezalova H, Lauerova L et al (1995) Conformational changes in p53 analysed using new antibodies to the core DNA binding domain of the protein. *Oncogene* **10**: 389–393

Yokomizo A, Mai M, Tindall DJ et al (1999) Overexpression of the wild type p73 gene in human bladder cancer. *Oncogene* **18**: 1629–1633

Zalcman G, Schlichtholz B, Trédaniel J et al (1998) Monitoring of p53 auto antibodies in lung cancer during therapy: relationship to response to treatment. *Clin. Cancer Res.*, **4**: 1359–1366

© 2001 Cancer Research Campaign 
